# Insect antimicrobial peptides: potential weapons to counteract the antibiotic resistance

**DOI:** 10.1007/s00018-021-03784-z

**Published:** 2021-02-17

**Authors:** M. D. Manniello, A. Moretta, R. Salvia, C. Scieuzo, D. Lucchetti, H. Vogel, A. Sgambato, P. Falabella

**Affiliations:** 1 Department of Sciences, University of Basilicata, Via dell’Ateneo Lucano 10, 85100 Potenza, Italy; 2 Spinoff XFlies S.R.L, University of Basilicata, Via dell’Ateneo Lucano 10, 85100 Potenza, Italy; 3 Department of Translational Medicine and Surgery, Università Cattolica del Sacro Cuore, Rome, Italy; 4 Department of Entomology, Max Planck Institute for Chemical Ecology, Hans-Knöll-Straße 8, 07745 Jena, Germany; 5 Centro di Riferimento Oncologico Della Basilicata (IRCCS-CROB), Rionero in Vulture (PZ), Italy

**Keywords:** Immune system, Antibacterial peptides, Resistant bacteria, Mechanism of action, Signaling pathways

## Abstract

Misuse and overuse of antibiotics have contributed in the last decades to a phenomenon known as antibiotic resistance which is currently considered one of the principal threats to global public health by the World Health Organization. The aim to find alternative drugs has been demonstrated as a real challenge. Thanks to their biodiversity, insects represent the largest class of organisms in the animal kingdom. The humoral immune response includes the production of antimicrobial peptides (AMPs) that are released into the insect hemolymph after microbial infection. In this review, we have focused on insect immune responses, particularly on AMP characteristics, their mechanism of action and applications, especially in the biomedical field. Furthermore, we discuss the Toll, Imd, and JAK-STAT pathways that activate genes encoding for the expression of AMPs. Moreover, we focused on strategies to improve insect peptides stability against proteolytic susceptibility such as D-amino acid substitutions, N-terminus modification, cyclization and dimerization.

## Introduction

### The antibiotic resistance as a global concern

Today, the identification of novel antibacterial therapeutics represents an auspicious perspective [1]. In fact, the inappropriate consumption and overuse of the first-line maintenance therapies have favored, in the last decades, an increasing selection of antibiotic-resistant pathogens. This phenomenon, together with the lack of availability of new molecules, represents real issues in health care [2, 3]. The multi-drug-resistant pathogens, such as ESKAPE (i.e. *Enterococcus faecium*,* Staphylococcus aureus*,* Klebsiella pneumoniae*,* Acinetobacter baumannii*,* Pseudomonas aeruginosa,* and *Enterobacter*) species, are considered practically resistant to most of the available antibiotics and have played a critical role in the growth of nosocomial infections [4, 5]. Moreover, the World Health Organization (WHO) has recently updated the priority list constituted of 12 bacterial pathogens against which there is a need to develop new antibiotics. The WHO list is divided into three categories according to the urgency of need for new antibiotics: critical, high, and medium priority. The category with a critical priority comprises the Gram-negative species *P. aeruginosa,* and *A. baumannii*, both carbapenem-resistant bacteria frequently associated with severe and often lethal diseases such as bloodstream infections and pneumonia. Several Gram-positive species constitute the high priority list, among which *S. aureus* methicillin-resistant (MRSA), and vancomycin-intermediate and resistant [6].

The increasing rate of antibiotic resistance represents a particularly challenging issue in the treatment of topical infections. Several complications such as chronic skin and soft tissue infections which can complicate the clinical course of ulcers, diabetic foot infections, post-surgical infections, and burn wounds are characterized by a progressive worsening of their clinical outcome, when antibiotic-resistant pathogens are involved. Likewise, the Gram-negative bacterium *A. baumannii* has been reported as responsible for a variety of antibiotic-resistant infections such as wound, skin, and urinary tract infections, but also pneumonia and bacteremia [7].

Bacterial infections of the lower respiratory tract, often related to bronchiectasis, represent an increasing and common chronic respiratory disease, associated not only with cystic fibrosis (CF) lung disease but also to chronic obstructive pulmonary disease. The clinical course of an antibiotic-resistant bronchiectasis can face, therefore, a progression of the health-condition worsening, due to the establishment of an infection-inflammation cycle uncontrollable by available drugs [8–10]. Lung infections associated with bronchiectasis may evolve to respiratory failure and death. Moreover, reduced quality of life and an increase in healthcare costs can worsen the patient compliance [11].

It is noteworthy that the successful management of bacterial infections is the product of combined actions of the host immune response and the administration of antibiotics. Hence, deficiencies of the host immune system and/or reduced efficacy of antibiotics due to the presence of resistant pathogens might, unfortunately, contribute to switching towards a persistent infection. Chronic bacterial infections are associated with increased morbidity and mortality from the infection itself as well as an increased risk of dissemination of disease which is a life-threatening condition difficult to treat, especially in the presence of antibiotic-resistant pathogens.

Therefore, along with the irresponsible use of antibiotics, the related resistance issue towards the most commonly used molecules represents a global concern [12]. The aim to find alternative drugs has demonstrated to be a real challenge, as well [13, 14].

### The biofilm issue

Bacterial pathogens have established various ways to defeat the host’s immune response so that bacterial virulence has been analyzed for decades. In nature, bacteria are physically grouped in clusters and embedded by extracellular polymeric substances [15]. In clinical settings, pathogen bacteria can effortlessly survive when colonizing surfaces (e.g. on wounds, scar tissue, medical implants), since sessile cells are less prone than planktonic to interact with the ordinarily used antimicrobials. Biofilms are bacterial communities embedded within an extracellular matrix and adherent to a surface. Biofilm formation is one of the main mechanisms of surviving, and it regards a wide range of microbes that commonly cause chronic infections [16]. One essential feature of biofilms is the intrinsic resistance of the bacterial community to the host immune system by decreasing efficacy of antibodies and antimicrobial peptides (AMPs) as well as phagocytic uptake within it thus hampering leukocyte-mediated killing. Moreover, the biofilm extracellular matrix component can partly limit the diffusion of antibiotics, thus, reducing their antimicrobial efficacy. The most relevant clinical biofilm-forming bacteria comprise the Gram-negative *A. baumannii*, *Escherichia coli*, *K. pneumoniae*, and *P. aeruginosa*, along with Gram-positive *S. aureus* and the less virulent *Staphylococcus epidermidis*.

Over 80% of chronic wounds are related to bacterial biofilms, in which the most commonly isolated pathogens are *S. aureus* and *P. aeruginosa* [17–23]. Humans are transporters of *S. aureus* infections; a range of 20–75% of humans demonstrated to be intermediate carriers. Several human body sites, such as nasal cavities, pharynx, perineum, and skin represent the primary sites for colonization. Commonly, the colonization by *S. aureus* causes no serious health problems in healthy individuals. However, the risk of developing *S. aureus* infection increases in the case of hospitalized patients with wounds, burns, and chronic ulcers [14]. *P. aeruginosa* is a ubiquitous bacterium that colonizes the natural environment near humans. Nevertheless, it represents a crucial but also one of the most resistant pathogens in CF lung disease [24]. Both representative species have been often associated with a biofilm mode of growth when isolated in the lower airways and portrayed as highly recalcitrant to the antibiotic treatments [25–27].

### Failure of the common pharmacological approaches

Multiple reasons for clinical failure can be mentioned when referring to antibiotic resistance. Poor pharmacokinetics of drugs in infection sites, or the bacterial phenotype of persistence, associated with the ability to survive in protected niches such as biofilms, foster the clinical failure. As mentioned above, specific bacteria may persist during the antibiotic treatment when drugs are administered at concentrations that should be lethal. Hence, this behavior may cause prolonged and recurrent infections [11]. Thus, antibiotic resistance is associated with higher medical costs, longer hospitalization, and increased mortality. To fight antibiotic resistance, a great effort has been devoted in the last decades to the development of new molecules, acting as antibiotics. Nevertheless, only a few new classes of antibiotics reached market availability in the last 3 decades, and others are still in human clinical trial. The clinical outcomes of resistant infections are also related to the host response to infection and to pathogen-derived toxic compounds. *S. aureus*, for example, can produce molecules able to counteract neutrophil’s action, i.e. preventing the adhesion to the blood vessels and their transmigration into the site of infection or eliciting cell death [28].

Regarding the skin infection management, the increasing issue related to resistance to the commonly used antibiotics mainly concerns the involvement of the deeper tissues which can cause the clinical worsening of wounds with clearly systemic infection risk. *S. aureus* is also associated with catheter-related bacteremia and intubation-related infections, such as pneumonia and bloodstream infections. Since the increasing resistance of bacterial pathogens often needs the use of more toxic agents to be counteracted, the antimicrobial therapy by topical application involves the use of not only suitable (e.g. considering toxic antibiotics) but also higher dosing as well [13, 29–31].

## Natural sources of antimicrobials

Screening of natural products has allowed the identification of some of the most active drugs. Biologically, active natural peptides can represent useful alternatives being characterized by high therapeutic efficacy, a low probability of resistance emerging in target cells, and limited side effects. To this aim, the exploitation of new compounds and the identification of their mechanisms of action is essential for further development. Antimicrobial peptides (AMPs), small molecules composed of 10–100 amino acid residues produced by all organisms, are attractive candidates for the design of new antibiotics because of their natural antimicrobial properties and a low propensity for the development of resistance [32]. Indeed, these natural peptides have retained their activity over the course of the evolution, triggering little or no resistance, and might represent a valuable alternative to classical drugs. Moreover, several evidence suggest that they can display anticancer activities characterized by a strong selectivity and efficacy on cancer cells so that many of them are also indicated as anticancer peptides (ACPs) [33–35]. Therefore, AMPs and ACPs are the focus of a large number of studies aimed at developing new antibiotics against multi-resistant bacteria (MDR) and new anticancer drugs AMPs are usually cationic and amphipathic and their structure includes both hydrophobic and hydrophilic moieties with a highly positive net charge (ranging from + 2 to + 9). They can be effective on a wide spectrum of microorganisms and can display powerful antimicrobial activities against antibiotic-resistant bacteria. Insects are an almost inexhaustible source of biologically active compounds. Natural products deriving from insects have been used for centuries in traditional medicine and still represent an essential source of healing substances in developing countries.

## Insects as natural sources of antimicrobials

### Overview

**Fig. 1:**
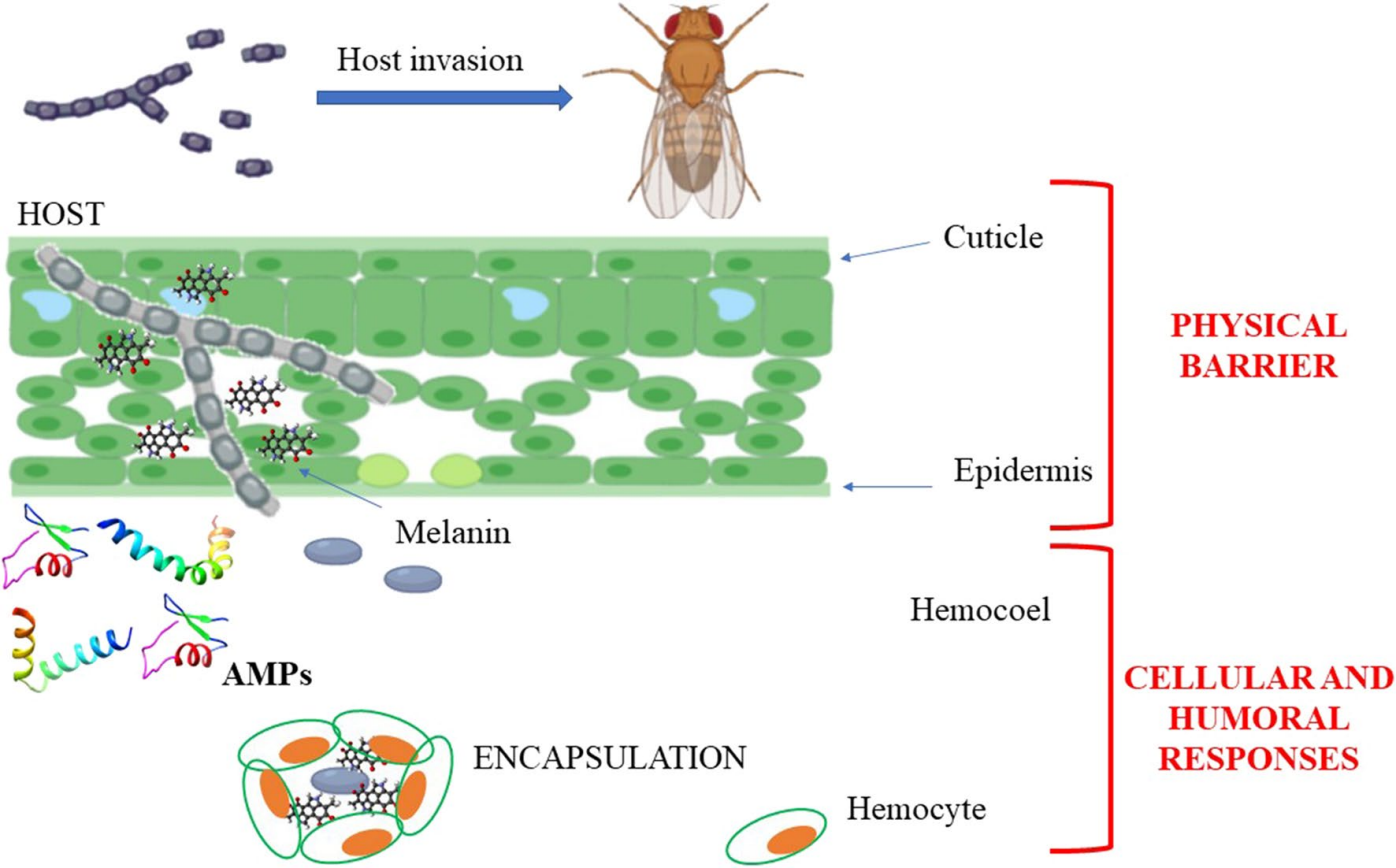
Schematic representation of insect immunity system. The first protections against the host invasion are physical barriers, including cuticle and epidermis. When pathogens succeed in overcoming these barriers, cellular and humoral immune responses are triggered, involving melanization, AMP production, and/or reaction mediated by hemocytes. Adapted from Lu and St. Leger, 2016, created with BioRender.com

Considering over one million described species, insects represent the largest class of organisms, due to their ability to adapt to recurrent changes and their resistance to a broad spectrum of pathogens [36, 37]. This resistance skill is related to their immune system, based exclusively on the innate immune response, which allows a broad and fast response to invading organisms [1, 38–40]. With the aim to prevent the entrance of pathogens within the hemocoel cavity, the first protection is represented by physical barriers such as the cuticle, the intestinal wall, and the tracheas (Fig. 1) [40, 41].

In recent years, an increasing number of insect AMPs have been proving useful in several applications concerning the pharmaceutical as well as the agricultural fields. Moreover, insect AMPs aroused great interest for their biomedical application thanks to the growing number of identified peptides that can inhibit human pathogens. AMPs susceptible pathogen bacteria include multidrug-resistant *E. coli*,* K. pneumoniae*,* Bacillus coagulans*,* Citrobacter freundii*,* Francisella tularensis*,* Streptococcus sanguinis*, and *S. aureus* [41–45]. Besides, some insect AMPs can also inhibit virus replication such as the two alloferons from the blowfly *Calliphora vicina*. These compounds have been demonstrated to be active against both human influenza viruses A and B [46]. Furthermore, melittin, peptide derivative from *Apis mellifera*, shows antiviral activity against herpes simplex virus 1 (HSV-1) [47]. Several fungi are also susceptible to insect AMPs including *Pichia pastoris*, *Aspergillus fumigatus*,* Cryptococcus neoformans*,* Botrytis cinerea*,* Fusarium *spp*.*,* Neurospora crassa,* and *Trichoderma viride* [48–50]. Given the increasing bacterial resistance to antibiotics, there is a great interest in verifying the AMPs suitability for the treatment of recalcitrant bacterial infections and killing of resistant bacteria. Several reports have highlighted that insect-derived AMPs can represent good candidates as alternatives to conventional antibiotics [51–53]. However, the treatments to inhibit pathogenic infections using cecropins, positively charged AMPs originally isolated from insects, for example, have suffered from some limitations. Indeed, they represent a target of human elastase produced by neutrophils, which are recruited during infections, or can be subjected to protease degradation [54, 55].

Insect AMPs represent a highly promising alternative to overcome medical problems associated with antibiotic resistance. Several studies have been performed using insect cecropins in the functionalization of biomaterials used in biomedicine, such as hydrogels and polyurethane surfaces [56, 57]. Moreover, cecropin expression in transgenic plants can confer resistance to bacterial and fungal pathogens [58, 59]. Transgenic expression of an insect cecropin (sarcotoxin-IA) and defensin (*Galleria mellonella* named gallerimycin) in tobacco also confers resistance to fungi [60].

### Organization of insect immunity system

All invertebrates including insects have a defense mechanism exclusively based on a powerful innate immune system, which allows a general and rapid response to different invading organisms [61]. The first protection against pathogens is represented by physical barriers such as the cuticle, the intestinal wall including the peritrophic membrane, and the tracheas [62]. If the foreign organisms pass through these defensive barriers, penetrating the hemocoel, the immune response is triggered. The innate immune system is conserved across all organisms comprising cellular responses and humoral responses. In insects, cellular immune responses are mediated by hemocytes, the cellular component of hemolymph responsible for nodulation, encapsulation and phagocytosis of invading pathogens. On the other hand, the haemocytes together with the fat body cells are also involved in the mechanisms of the humoral response that includes AMP synthesis, the enzymatic cascade that regulates the activation of hemolymph coagulation and melanization, and the production of reactive oxygen (ROS) and nitrogen (RNS) species [63].

The clear separation between humoral and cellular response is more conventional than functional; some of the humoral factors regulate the activity of haemocytes and, at the same time, haemocytes are the source of several molecules involved in the humoral response. Furthermore, they often share the same signal transduction pathways, even if activated by different stimuli [38, 64].

Among the humoral immune response in insects the production of melanin, a highly toxic phenolic biopolymer is involved both in the defense against pathogens and in the repair of cuticular wounds to prevent the loss of hemolymph [65]. Melanogenesis is regulated by the pro-phenoloxidase (proPO) system, a cascade of serine proteases and inhibitors of serine proteases that finely control the activation of proPO, the precursor of phenoloxidase (PO), after the recognition of an external elicitor [66]. Many data have shown that proPO is synthesized and accumulated in haemocytes and, when necessary, released by a lytic process which does not necessarily lead the cell to death [67]. Other studies have shown that proPO is localized on the surface of hemocytes. This localization could facilitate the deposition of melanin directly on the foreign agent [68, 69]. Melanin is a very toxic compound and its systemic diffusion would be extremely harmful for the insect: the localization of its synthesis is essential to ensure the survival of the insect during the activation of melanogenesis [70]. Melanogenesis also generates cytotoxic intermediates, such as quinones and semiquinones, which favor the synthesis ROS and RNS. Moreover, these intermediates, alone or in combination with ROS and RNS, are cytotoxic molecules that participate in the elimination of the pathogen [71].

Cellular immune response is mediated by hemocytes. In most species of different orders, such as *Lepidoptera*, *Diptera* (except *Drosophila*), *Orthoptera*,* Blattoidei*,* Coleoptera*,* Hymenoptera*, *Hemiptera*, and *Collemboli*, the hemocytes are differentiated into granulocytes, plasmatocytes, spherulocytes, and oenocytoids [64, 72]. In *Lepidoptera*, granulocytes and plasmatocytes, which represent more than 50% of the circulating hemocytes, show adhesive ability. Plasmatocytes are also involved in the production of AMPs as well as in the release of extracellular matrix components [39]. The other two components of hemocytes, the spherulocytes, which carry cuticle components, and the oenocytoids, containing precursors of the activation cascade of the PO, have not any adhesive ability [39].

**Fig. 2:**
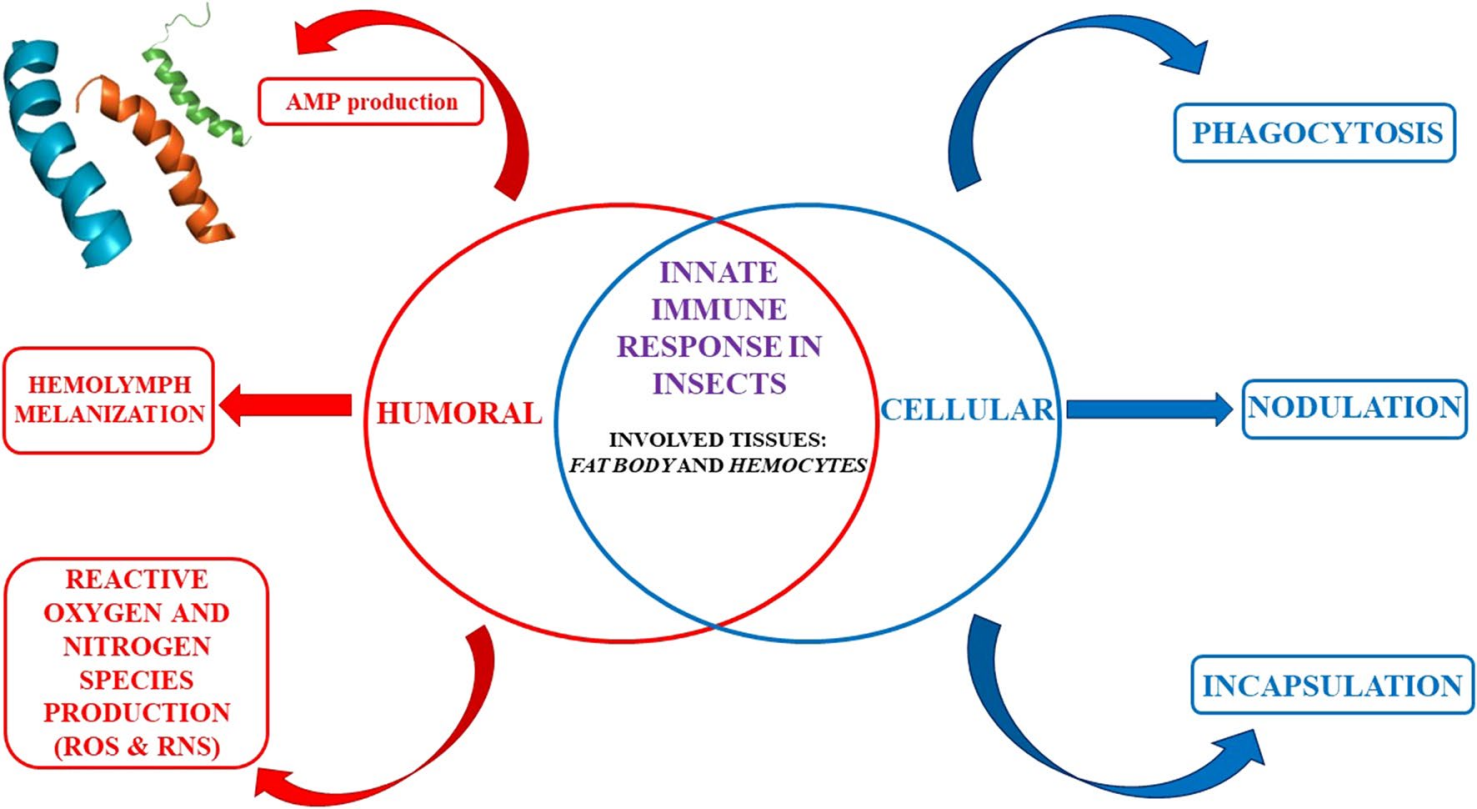
Insects innate immune response can be humoral or cellular. Humoral immunity consists of AMPs production by the fat body and/or hemocytes; hemolymph melanization and production of the reactive oxygen and nitrogen species. Cellular immunity consists of phagocytosis, nodulation and encapsulation processes. Phagocytosis determines the internalization of foreign agents by the hemocytes and the transport of the phagocyte material into the phagosomes where it is degraded. Nodulation occurs when bacteria are too much to be incorporated by a single hemocyte. Indeed, several hemocytes together recognize and surround foreign agents. In the encapsulation process, hemocytes create a capsule made up of several cell layers that undergoes melanization. Inside the capsule, the pathogenic organism is killed by asphyxiation and/or production of cytotoxic free radicals [64, 72]

Phagocytosis, mediated by hemocytes, includes the recognition and encapsulation of foreign agents through modifications of the hemocyte cytoskeleton and ends with the transport of the phagocyte material into the phagosomes where it is completely degraded thanks to the action of hydrolase, ROS and nitric oxide [72] (Fig. 2). In most insect orders, both granulocytes and plasmatocytes are responsible for phagocytosis while in *Drosophila melanogaster* this role is played by plasmatocytes alone [64, 72]. During the immune response, the nodulation process is activated when a large number of bacteria cannot be phagocytized by a single hemocyte. In this process, several kinds of hemocytes recognize and surround microorganisms, thus forming complexes that may or may not undergo melanization [73] (Fig. 2). In the encapsulation process, hemocytes adhere to surfaces of invading agents that are too big to be phagocytized, such as parasites, protozoa or nematodes consequently forming a capsule, made up of several cell layers, that undergoes melanization. Inside the capsule, the pathogenic organism is killed by asphyxiation or by the production of cytotoxic free radicals [72] Granulocytes and plasmatocytes are involved in capsule formation in *Lepidoptera* [64, 72] while this role is played by plasmatocytes and lamellocytes in *D. melanogaster* [74] (Fig. 2).

### Activation of the insect immune response

The triggering of the insect immune response is generated only when the exogenous agent is recognized, identifying specific and preserved molecules located on the pathogen surface the defined as pathogen-associated molecular patterns (PAMPs) [75]. PAMPs are molecular components potentially present in all microorganisms but absent in higher organisms. Examples of PAMPs comprise Gram-positive lipoteichoic acid and peptidoglycan, Gram-negative bacteria lipopolysaccharide (LPS), and fungi β-1,3-glucan [76]. These non-self-molecules are recognized by specific receptors (named pattern-recognition proteins, PRPs), which can be both humoral and cellular. Immunolectins, peptidoglycan recognition proteins (PGRPs), and Gram-negative binding proteins (GNBPs) are proteins circulating in the hemolymph and capable of recognizing specific antigens [40]. Peptidoglycan-recognition protein LC (PGRP-LC) and integrins, on the other hand, are receptors found on the surface of immune cells, which, respectively, recognize surface components of Gram-negative bacteria and the RGD motif (Arg-Gly-Asp) [36, 40]. The latter is found in the proteins of the extracellular matrix and in some soluble proteins such as collagen, fibronectin, and laminin. The binding of integrins to the RGD motif, for example, represents the first step for the recognition of exogenous agents. Furthermore, it is involved in bacterial phagocytosis or in the encapsulation process [40]. When the receptors, both humoral and cellular, bind to pathogen-associated molecules, specific immune responses are triggered based on the type of invader [1, 39, 40]. The humoral immune response includes the production of AMPs, the enzymatic cascade that regulates the activation of hemolymph coagulation, melanization, and the production of reactive oxygen as well as nitrogen species (often indicated as ROS and RNS, respectively) [71] (Fig. 2).

Due to the relevance of AMP function in insects, in the following section, we focused on insect AMPs with a special emphasis on their classification, overviewing their structural and functional characteristics, along with reviewing the signaling pathways which activate the encoding AMP genes and their mechanism of action.

## Overview of insect antimicrobial peptides

Insects can interact with the ecosystem using chemical substances. Besides, a variety of species can contribute to investigate the potential of new molecules [77]. Although it is possible to find smaller or larger peptides in nature, AMPs comprise small molecules whose amino acid composition ranges from 12 to 50 amino acids [53].

AMPs are involved in several defence-related processes such as the binding and the neutralization of endotoxins, the modulation of the immune responses to infection, and the pathogens killing [78]. The first insect AMP, the cecropin, was identified in the 1980 from the pupae of *Hyalophora cecropia* [42, 79]. AMPs show a wide range of antibacterial, antiviral, anticancer, and antifungal activity [80–82]. In the last few years, the number of identified insect peptides has considerably increased, thanks to the published insect genome, transcriptome, and proteomic datasets (OMIC analysis). Mass spectrometry methodologies are adopted for the analysis of insect hemolymph, extracted from bacteria-induced larvae [83]. Both peptides and proteins have been considered as a promising choice to treat various diseases. It is now known that the adoption of AMPs is a promising alternative to substitute or support the current antimicrobial approach. Moreover, 3180 AMPs have been identified from different kingdoms, among which bacteria (i.e. 355 bacteriocins), fungi (20 AMPs), plants (352 AMPs), and animal sources (2356 AMPs) [84] (http://aps.unmc.edu/AP/). So far, the Antimicrobial Peptide Database is currently reporting 311 out of the 3180 insect-derived AMPs [84]. Surely, the OMIC analysis can also contribute to increasing the number of peptides or proteins isolated by insects that could have antimicrobial activity and become new potential AMPs [85].

Most insect AMPs are cationic molecules due to the presence of basic residues with activities against bacteria. According to their amino acid sequences and structures, AMPs can be classified in four different groups: cysteine-rich peptides (e.g. defensins), the α-helical peptides (e.g. cecropins), glycine (Gly) -rich proteins (e.g. attacins), and proline-rich peptides (e.g. drosocins) [86, 87]. Number of AMPs in insects can widely vary among species: for example, 57 putatively active peptides were identified in *Hermetia illucens*, while very few peptides were identified in aphids [88, 89]. *H. illucens* is one of the most promising sources for AMPs, as the larval instar feed on vegetal and animal decaying organic substrates. Larvae are capable of producing several AMPs, which protect the insect from the pathogens in the substrate and are able to restore substrate health conditions, reducing the bacterial load of pathogenic species such as *E. coli* and *Salmonella enterica* [90, 91]. Recently a stomoxynZH1 from *H. illucens* was cloned and expressed in bacterial cells and tested against different bacterial and fungal strains, resulting in inhibition of *S. aureus* and *E. coli* (growing bacteria), as well as *Rhizoctonia solani* and *Sclerotinia sclerotiorum* (Lib.) de Bary (fungi) [92].

### Defensins (cysteine-rich AMPs)

Defensins are small cationic peptides due to the presence of basic amino acids, particularly arginine [93]. They consist of about 34–51 residues and contain six conserved cysteines (Cys) which form three intramolecular disulfide bridges. Insect defensins have been identified in several insect orders such as Hemiptera, Coleoptera, Diptera, Hymenoptera, and Lepidoptera, but also in the ancient order of Odonata, suggesting that they might derive from a common ancestor gene [94].

**Fig. 3:**
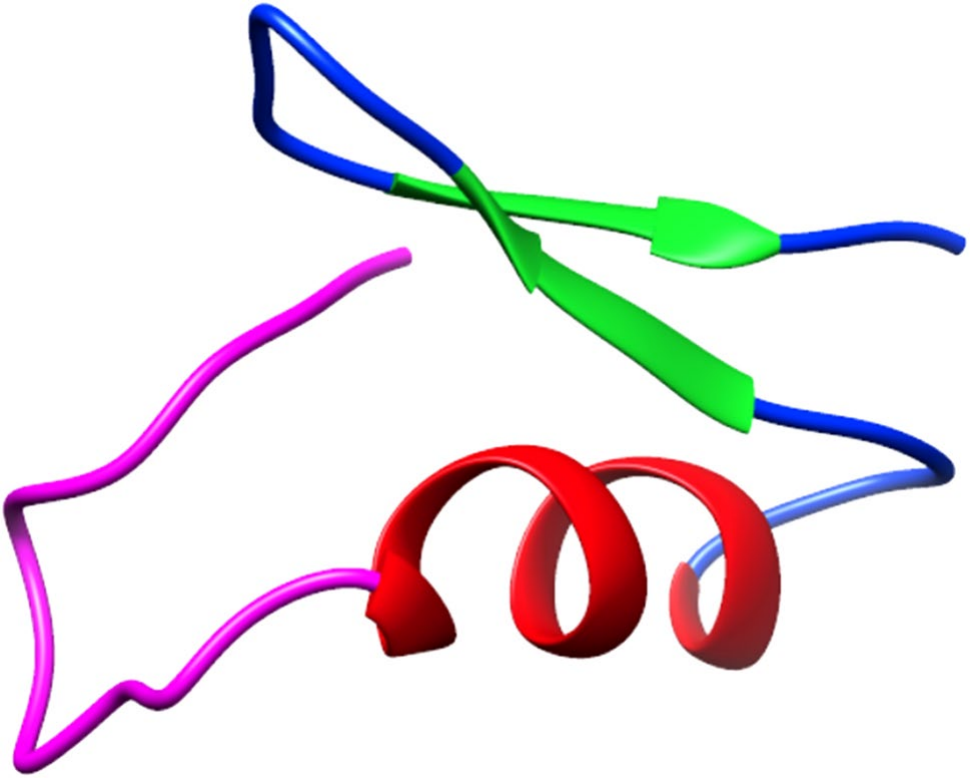
Structural representation of lucifensin, a defensin antimicrobial peptide identified in *Lucilia sericata*, obtained from the Protein Data Bank [95]. The N-terminal loop is shown magenta; the α-helix region in red and the antiparallel β-sheet in green. The image has been generated with UCSF CHIMERA software [98]

From a structural point of view, defensins show an N-terminal loop, an α-helix, followed by an antiparallel β-sheet, as shown in Fig. 3 for the defensin lucifensin (2LLD, PDB code) from *Lucilia sericata* (ATCDLLSGTGVKHSACAAHCLLRGNRGGYCNGRAICVCRN) [95–98].

**Fig. 4:**
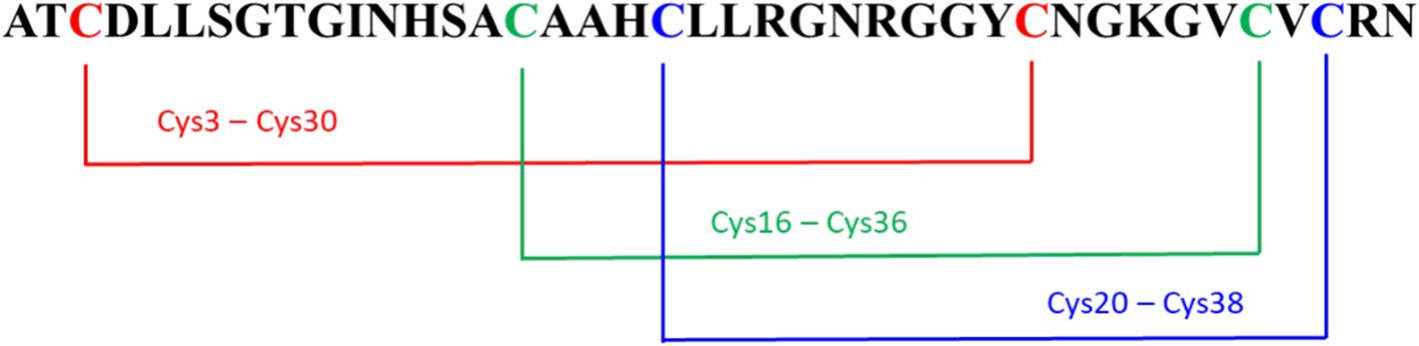
Disulfide bonds amongst Cys  of insect Defensins A from *Protophormia terraenovae*

**Fig. 5:**
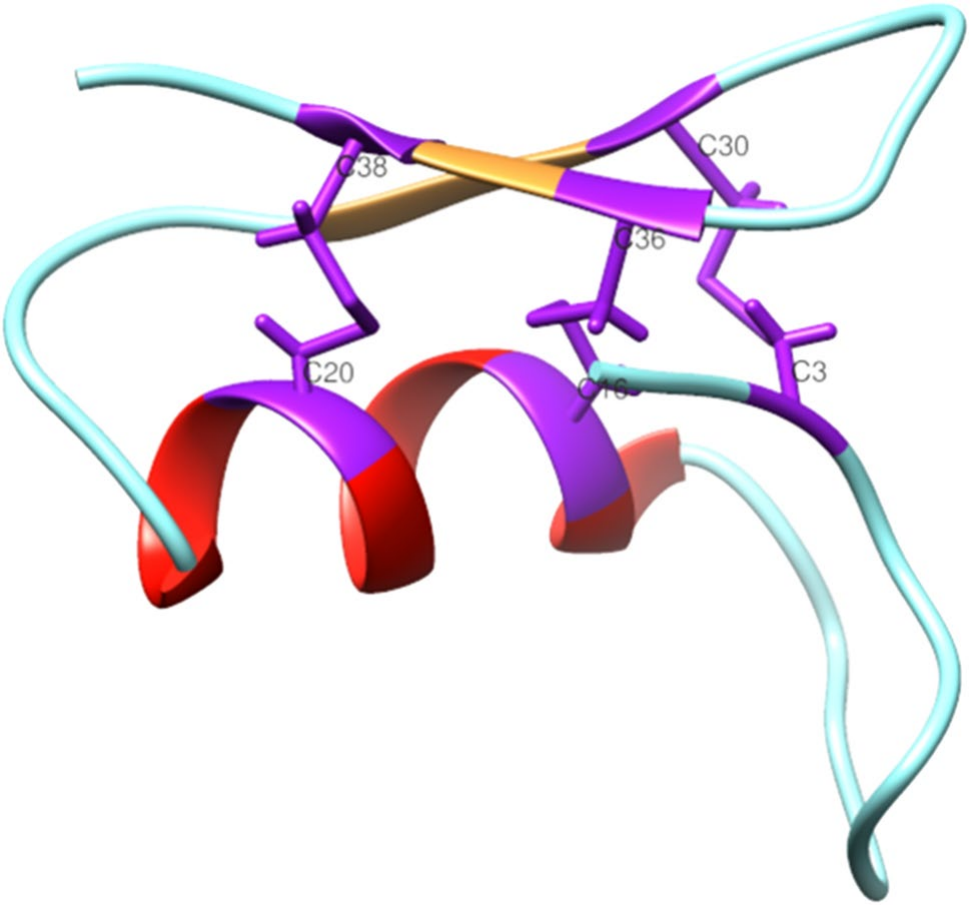
Structural representation of disulfide bonds in lucifensin. The loop is shown in cyan, the α-helix region in red, and the antiparallel β-sheet in orange while the cysteine residues, and the disulfide bonds are in purple. The image has been generated using UCSF CHIMERA software [98]

Two intramolecular disulfide bonds connect the β-sheet and the α-helix, forming a Cys-stabilized alpha beta (CSαβ) structure [97]. Considering the insect defensins, the Cys  are linked as Cys1—Cys4, Cys2—Cys5, and Cys3—Cys6 [99]. For example, Defensin A sequence from *Protophormia terraenovae* is shown in Fig. 4, and Fig. 5.

Insect defensins are particularly active against Gram-positive bacteria such as *S. aureus*,* Bacillus subtilis*,* Micrococcus luteus*, and *Bacillus megaterium*. Nevertheless, some of them have also shown antimicrobial activity against Gram-negative bacteria such as *E. coli* [100, 101]. In Table 1, the major insect defensins with reported antimicrobial activity are listed.

**Table 1:** Examples of insect defensins with reported antimicrobial activity

Peptide (species**)**	SwissProt accession number	Antimicrobial activity	Reference
Defensin (*Phlebotomus duboscqi*)	P83404	Gram-positive bacteria	[102]
Tenecin 1 (*Tenebrio molitor*)	Q27023		[103]
Defensin (*Bombus pascuorum*)	P81462	Gram-positive and Gram-negative bacteria	[104]
Coprisinc (*Copris tripartitus*)	A9XFZ7		[105]
Defensin 1 (*Acalolepta luxuriosa*)	Q9BK52		[106]
Defensin A (*Anomala cuprea*)	P83669	Gram-positive bacteria	[107]
Defensin B (*Anomala cuprea*)	P83668	Gram-positive and Gram-negative bacteria	
Defensin (*Calliphora vicina*)	C0HJX7	Gram-positive bacteria	[108]
Royalisin (*Apis mellifera*)	P17722		[109]
Defensin (*Pyrrhocoris apterus*)	P37364	Gram-positive and Gram-negative bacteria	[110]
Defensin (*Oryctes rhinoceros*)	O96049	Gram-positive bacteria	[111]

### Cecropins (α-helical AMPs)

**Fig. 6:**
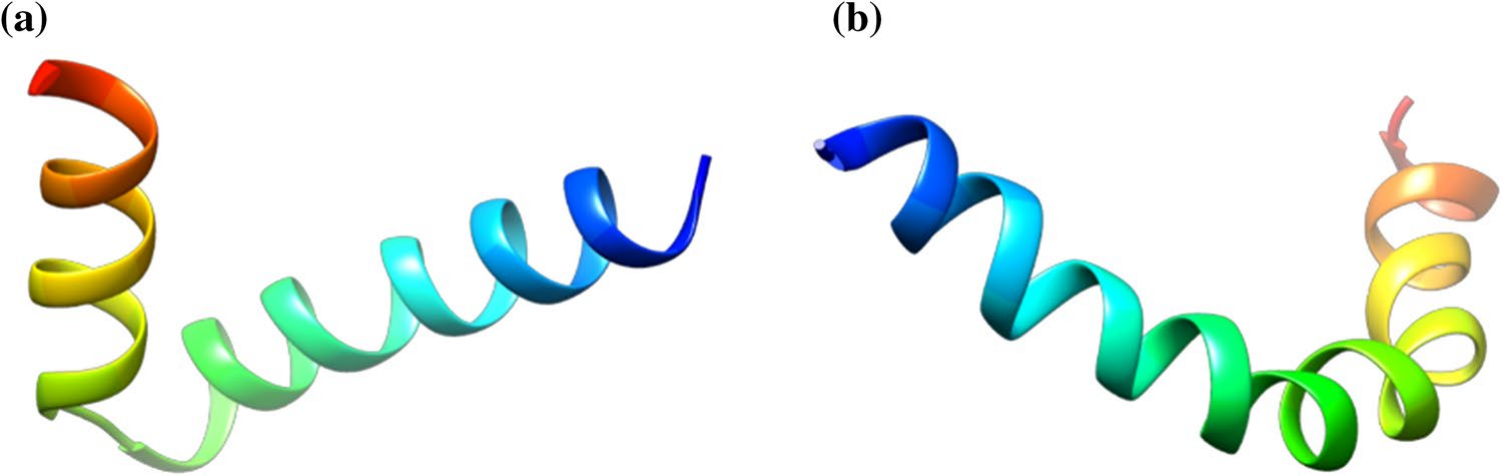
Structural representation of (**a**) papiliocin, identified in *Papilio xuthus* insect and (**b**) GK cecropin-like peptide from *Aedes aegypti*, obtained from the Protein Data Bank [95]. Images have been generated with UCSF CHIMERA software [98]

AMPs belonging to the cecropin family represent the most abundant linear α-helical AMPs in insects [38]. They were isolated for the first time from hemolymph of the lepidopteran *Hyalophora cecropia*. Before maturation, insect cecropins are composed by a range between 58 and 79 amino acids. The active forms contain between 34 and 55 residues and are mainly active against Gram-negative bacteria, and, to a lesser extent, against Gram-positive bacteria [112, 113]. It has been also demonstrated that some cecropins can exhibit (i) antifungal activity, (ii) a low toxicity against normal mammalian cells, and (iii) a weak, or absent in some cases, hemolytic effect against mammalian erythrocytes [114]. Moreover, most cecropins are subjected to amidation of the C-terminus, a post-translational modification that increases their antimicrobial activity [81]. Circular dichroism analyses demonstrated that in aqueous solution, cecropins assume a random coiled structure. However, upon the interaction with microbial membranes, cecropins adopt a α-helical conformation [115, 116]. In Fig. 6a, b, the structures of papiliocin (2LA2, PDB code) from *Papilio xuthus*, and GK cecropin-like peptide (2MMM, PDB code) from *Aedes aegypti*, respectively, are shown.

Several insect cecropins have been studied so far from both a structural and a biological point of view, evaluating their in vitro activity. For example, cecropin A has a stabilized α-helical structure and has been shown to reduce both NADP^+^ and glutathione levels, inducing oxidative stress by forming ROS, but its mechanism of action is still unknown [117, 118].

Cecropin A shows activity against the fungus *Beauveria bassiana* in silkworm larvae [119] but cecropin B, a linear cationic peptide, shows the highest and wide antibacterial activity among the cecropins family [120]. It has been reported that cecropin B decreases the bacterial load of *E. coli* and the concentration of plasma endotoxin it has exhibited antifungal activity against *Candida albicans* [115, 121]. Some cecropins also show anti-inflammatory activity [122, 123]. Inflammation is a protective response of a tissue triggered by pathogen infection and involved in the reparative processes [124]. In Table 2, the major insect cecropins with reported antimicrobial activity are listed.

**Table 2:** Examples of insect cecropins with reported antimicrobial activity. All the listed cecropins are active against both Gram-positive and Gram-negative bacteria

Peptide (species)	SwissProt accession number	Antimicrobial activity	Reference
Cecropin A (*Spodoptera litura*)	Q9XZG9	Gram-positive and Gram-negative bacteria	[125]
Cecropin B (*Spodoptera litura*)	Q9XZH0		
Stomoxyn (*Stomoxys calcitrans*)	Q8T9R8		[126]
Cecropin A (*Hyalophora cecropia*)	P01507		[38]
Cecropin B (*Hyalophora cecropia*)	P01508		[113]
Cecropin D (*Hyalophora cecropia*)	P01510		[127]
Cecropin A (*Bombyx mori*)	Q27239		[113]
Cecropin B (*Bombyx mori*)	P04142		
Cecropin D (*Bombyx mori*)	O76146		
Papiliocin (*Papilio xuthus*)	D8L127		[128]
Cecropin B (*Antheraea pernyi*)	P01509		[129]
Cecropin B (*Antheraea pernyi*)	P01511		

### Attacins

Attacins are Gly-rich proteins, first purified from the hemolymph of *H. cecropia* bacteria-immunized pupae. Attacins are produced as pre-pro-proteins with a signal peptide, a pro-peptide, an N-terminal attacin domain and two Gly-rich domains, called G1 and G2 domains [130].

They can be divided in two groups: the acidic (i.e. attacin E, and F), and basic (i.e. attacins A–D) attacins [131]. Even though attacins are encoded by two different genes [132] and they have been identified in lepidopteran and dipteran species [133–137], they show high similarity in the amino acid sequences.

They are mostly active against Gram-negative bacteria, particularly *E. coli* and some Gram-positive bacteria. For example the attacin peptide from *Spodoptera exigua*, is active against *E. coli* and *Pseudomonas cichorii* but also against Gram-positive *Bacillus subtilis* and *Listeria monocytogenes* [138, 139].

### Glycine-rich AMPs

Gloverins are Gly-rich peptides identified in the *Lepidoptera* insect order and synthetized as pre-pro-proteins [140]. They are basic molecules, and, in aqueous solution, they take a random coil structure, assuming an α-helical structure in a hydrophobic environment [141]. The first gloverin peptide was purified from the hemolymph of *Hyalophora gloveri* pupae [141]. Gloverin peptides are mostly active against Gram-negative bacteria, particularly *E. coli*, but some of them exhibit antimicrobial activity against Gram-positive bacteria, fungi, and viruses [140, 142]. Gloverin peptide identified in *Manduca sexta*, although exhibiting activity against the Gram-positive bacteria *Bacillus cereus*,* Saccharomyces cerevisiae*, and *C. neoformans*, show no activity against *E. coli* [140].

**Fig. 7:**
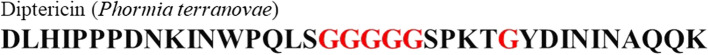
Sequence of a glycine-rich peptide, Diptericin from *Phormia terraenovae.* Highlighted in red the glycine residues

Diptericins are another class of Gly-rich peptides. Diptericins A–C have been isolated from immunized larvae of *Phormia terraenovae* (Fig. 7), in *Sarcophaga peregrina* and in *D. melanogaster* [143–145]. Prolixicin, a 21 amino acid peptide, has been isolated from *Rhodnius prolixus* and it is released by midgut tissues after the hemolymph bacterial infection [39].

### Proline-rich AMPs

**Fig. 8:**
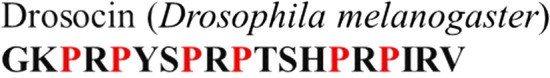
Sequence of a proline-rich peptide, Drosocin from *Drosophila melanogaster.* Highlighted in red the proline residues

Proline-rich AMPs have a high content of Pro residues. Among them, Lebocins are proline-rich peptides first isolated from the hemolymph of *Bombyx mori* immunized with *E. coli* [146]. Lebocins show antimicrobial action against Gram-positive and Gram-negative bacteria, as well as against some fungi. They were identified in *B. mori*, and require the O-glycosylation for their full activity mainly against *Acinetobacter sp*. and *E. coli* [146]. Other proline-rich AMPs have been identified, such as drosocin, produced by *D. melanogaster* (Fig. 8). Drosocin is an O-glycosylated 19 amino acid peptide and shows a significant sequence homology with Apidaecin IB peptide, isolated from *A. mellifera* [147, 148]. Apidaecins are involved in the honeybee humoral defense against microbial invasion [148].

Moreover, a 26-residue proline-rich immune-inducible linear peptide called Metchnikowin, has been identified in *D. melanogaster*, by Levashina et al. [149]. However, this peptide is not active against Gram-negative bacteria, whereas it exhibits antimicrobial activity against Gram-positive bacteria and fungi. Concerning the antifungal activity, Metchnikowin targets the iron-sulfur subunit (SdhB) of succinate-coenzyme Q reductase [150] and it interacts with the fungal enzyme (1,3)-glucanosyltransferase Gel1 (FgBGT) which is involved in fungal cell wall synthesis [150].

## Signaling pathways and mechanisms of action

### AMP gene activation—Toll, Imd, and JAK-STAT pathways

**Fig. 9:**
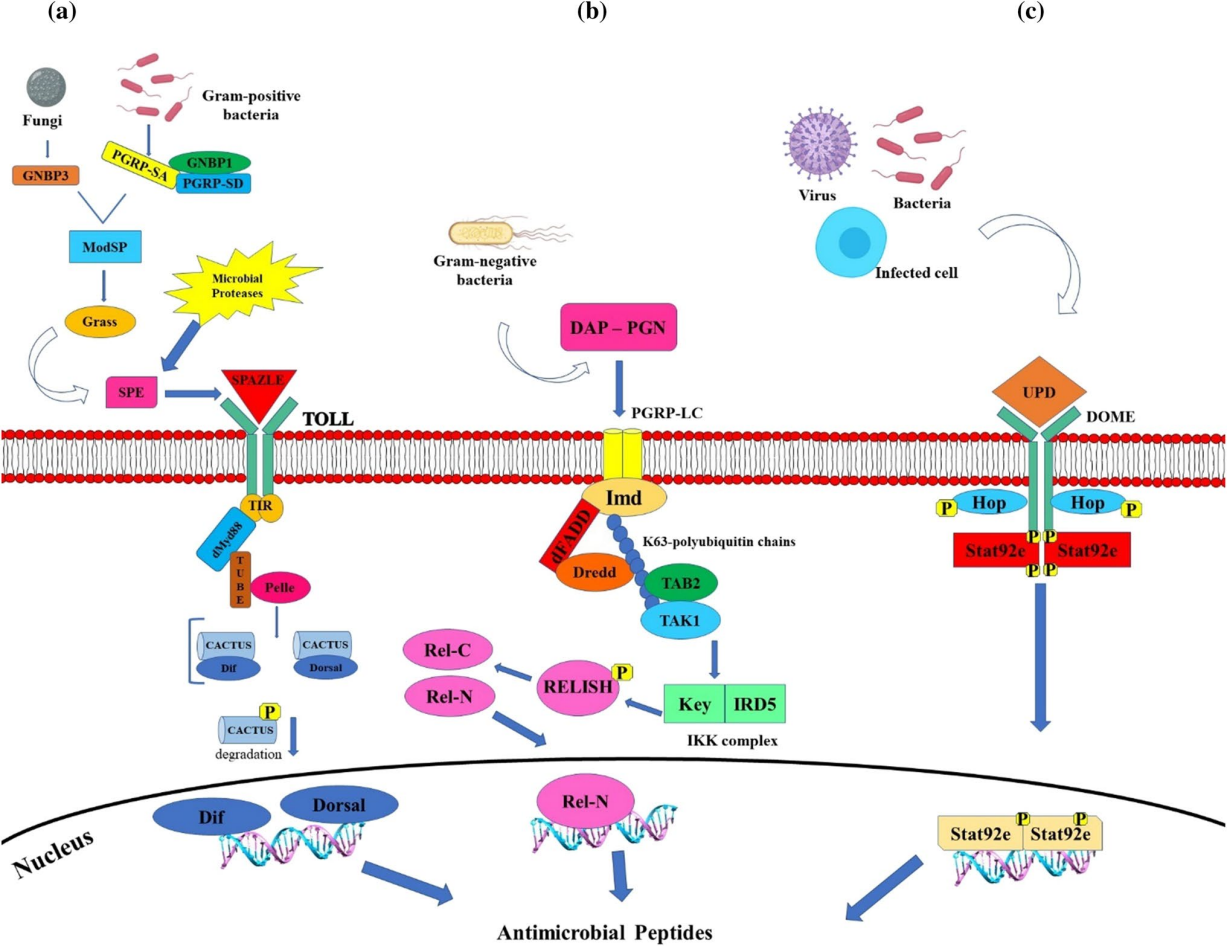
Schematic representation of Toll (**a**), Imd (**b**), and JAK-STAT (**c**) signaling pathways. In insects, the Toll pathway is mainly involved in fungi and Gram-positive bacteria detection. Pathogen recognition peptidoglycan recognition proteins (PGRP) activate a serine proteases cascade, involving ModSP and Grass proteins, which in turn, cleaving the inactive form of Spätzle protein, switch on the molecule. These interactions initiate protease cascades. Spätzle activates the dimer Toll receptor, which, in turn recruits cytoplasmic proteins (dMyD88, Tube, and Pelle) involved in the activation of Cactus signaling. In normal cellular condition, Cactus protein is coupled with the Nuclear Factor kappa B (NF-κB) transcription factors Dorsal-related immunity factor (DIF) and Dorsal, but following the Toll pathway activation, it is phosphorylated, detached from DIF and Dorsal and degraded. Then, both DIF and Dorsal can translocate in the nucleus and induce the transcriptional regulation of specific AMP genes (A) [160]. The insect Imd signaling pathway is activated following the binding between PGRP-LC and meso-diaminopimelic acid (DAP)-type peptidoglycan of Gram-negative and some Gram-positive bacteria. The Imd protein is activated following the cleavage by the Fas-associated death domain (FADD) and the death related ced-3/Nedd2-like caspase (DREDD). The K63‐polyubiquitin chains help to link this complex with TAK1 and TAB2 proteins that, in turn, act on the IKK complex, which phosphorylates the NF‐kB‐like nuclear factor Relish. Consequently, TAK1 and TAB2 proteins are activated, that in turn, act on the IKK complex, composed of Immune Response Deficient 5 (IRD5) and Kenny (Key). This activated complex cleaves Relish. In this way, the Rel DNA-binding domain is released from the C-terminal ankyrin-repeat/IκB-like domain, and translocates to the nucleus inducing specific AMP genes transcription (B) [160]. In insect, JAK/STAT pathway is activated when the cytokine receptor, Domeless (Dome), bind the Unpaired (Upd) cytokines which induces the JAK tyrosine kinase Hopscotch (Hop) to phosphorylate itself and the Dome cytoplasmic component. Simultaneously, the signal transducer and activator of transcription at 92E (Stat92e) bind to the phospho tyrosines on Dome, and they are phosphorylated by Hop. Phosphorylated Stat92e separates itself from the receptor, dimerize and relocate into the nucleus, where it induces the transcription of Thioester-containing protein genes (Teps) and Turandot (Tot) genes. Proteins derived from the transcription of these genes are involved in phagocytosis and melanization processes [160, 161]

Several signaling molecules are activated after detection of foreign microorganisms by pattern-recognition receptors. Among these, the main pathways are the Immune Deficiency (Imd), the JAK-STAT, and the Toll pathways, which have been well described in *D. melanogaster* (Fig. 6) [151–153]. Antigens of both Gram-positive bacteria and fungi can induce the Toll pathway by activating cellular immunity (Fig. 9a) [153]. Afterward, the signaling pathways involved in humoral immune responses are activated, leading to the release of AMPs, such as drosomycin, by the fat body [39]. The Toll pathway activates the nuclear factor κB (nuclear factor kappa-light-chain enhancer of activated B cells—NF‐κB) reacting in response to stress stimuli, such as in the presence of bacterial or viral antigens [153, 154]. The transmembrane receptor Toll is activated by the extracellular cytokine‐like polypeptide, called Spätzle, previously cleaved by serine protease cascades that, in turn, is triggered by the recognition of foreign agents [155]. Specifically, the Toll activation is mediated by peptidoglycan recognition proteins (PGRPs), Gram‐negative binding protein (GNBP) 1 in the case of Gram‐positive bacterial infection, whereas Toll activation is mediated by GNBP 3 in the case of fungal infections [156, 157]. Toll signaling is activated when Spätzle binds the Toll receptor (Fig. 9a) [158]. The dimerization of the intracytoplasmic TIR (toll-interleukin receptor) domains consequently starts, leading then to the binding of the adaptor protein Myeloid differentiation primary response 88 (MyD88) [153]. This protein binds the adaptor protein, Tube, which recruits the protein kinase Pelle for its autophosphorylation and phosphorylation and degradation of an IκB inhibitor, Cactus. The NF‐κB transcription factors Dorsal or Dif are then translocated into the nucleus where they activate the transcription of AMPs [159].

Concerning the infection signaling by Gram-negative bacteria, the Imd signaling pathway is activated when the PGRP‐LC receptors bind meso‐diaminopimelic acid (DAP)‐type peptidoglycan 2 (Fig. 9b). Imd binds to the Fas‐associated protein with death domain (FADD), while the caspase called DREDD (FADD‐death‐related ced‐3/Nedd2‐like protein) is recruited to cleave the Imd protein, which is then activated by K63‐ubiquitination [163, 164]. The K63‐polyubiquitin chains recruit TAK1 (transforming growth factor beta (TGF‐β)‐activated kinase 1), which activates the IKK complex involved in the phosphorylation of the NF‐κB‐like nuclear factor Relish. After Relish cleavage and phosphorylation, it reaches the nucleus where it activates transcription of specific AMPs, such as diptericin (Fig. 9b) [165].

In the Janus kinase‐signal transducer and activator of transcription (JAK‐STAT), JAKs are activated after the binding of a cytokine to its receptors and phosphorylate-specific tyrosine residues on the cytoplasmic part of the receptor and these residues then bind to STAT molecules [160, 166] (Fig. 9c). The STAT tyrosine residues are then phosphorylated by JAKs, leading to dimers formation and to the translocation into the nucleus, where they bind the promoters of their target genes [167]. In *D. melanogaster*, the JAK‐STAT pathway ligands consist of three cytokine‐like proteins called unpaired (upd), upd2 and upd3 [146]. The Dome receptor [168] binds to a single JAK molecule, hopscotch (hop) [169], and one STAT transcription factor, Stat92E for the induction of immune response genes [170].

However, the humoral immune response in *D. melanogaster* is principally controlled by the Toll and Imd pathways leading to the production of AMPs [153].

### Insect AMP mechanism of action

**Fig. 10:**
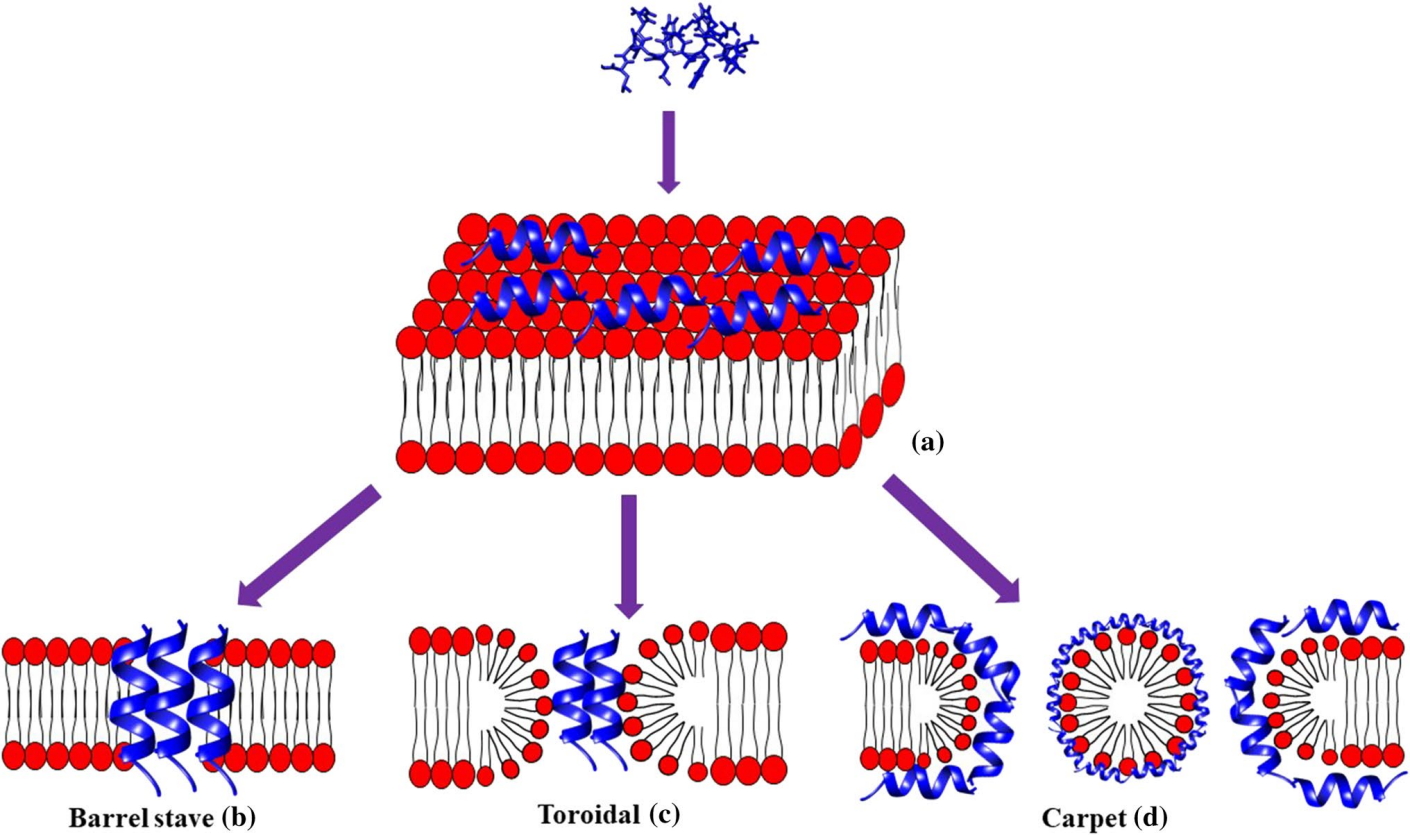
Schematic representation of AMP interaction with the bacterial membrane. Membranolytic mechanisms begin with adsorption of AMP on target cell membrane (**a**). In the barrel-stave model peptides permeate through the bilayer (**b**); in the toroidal pore mechanism, peptides interact with the head groups of the lipids, induce the bilayer curvature and perpendicularly insert into the membrane bilayer (**c**); in the carpet model, peptides cover all the membrane the membrane, the peptide non-polar side chains bind the membrane hydrophobic core while the polar residues with the lipid phosphates, forming micelles with the fragmented membrane (**d**)

**Fig. 11:**
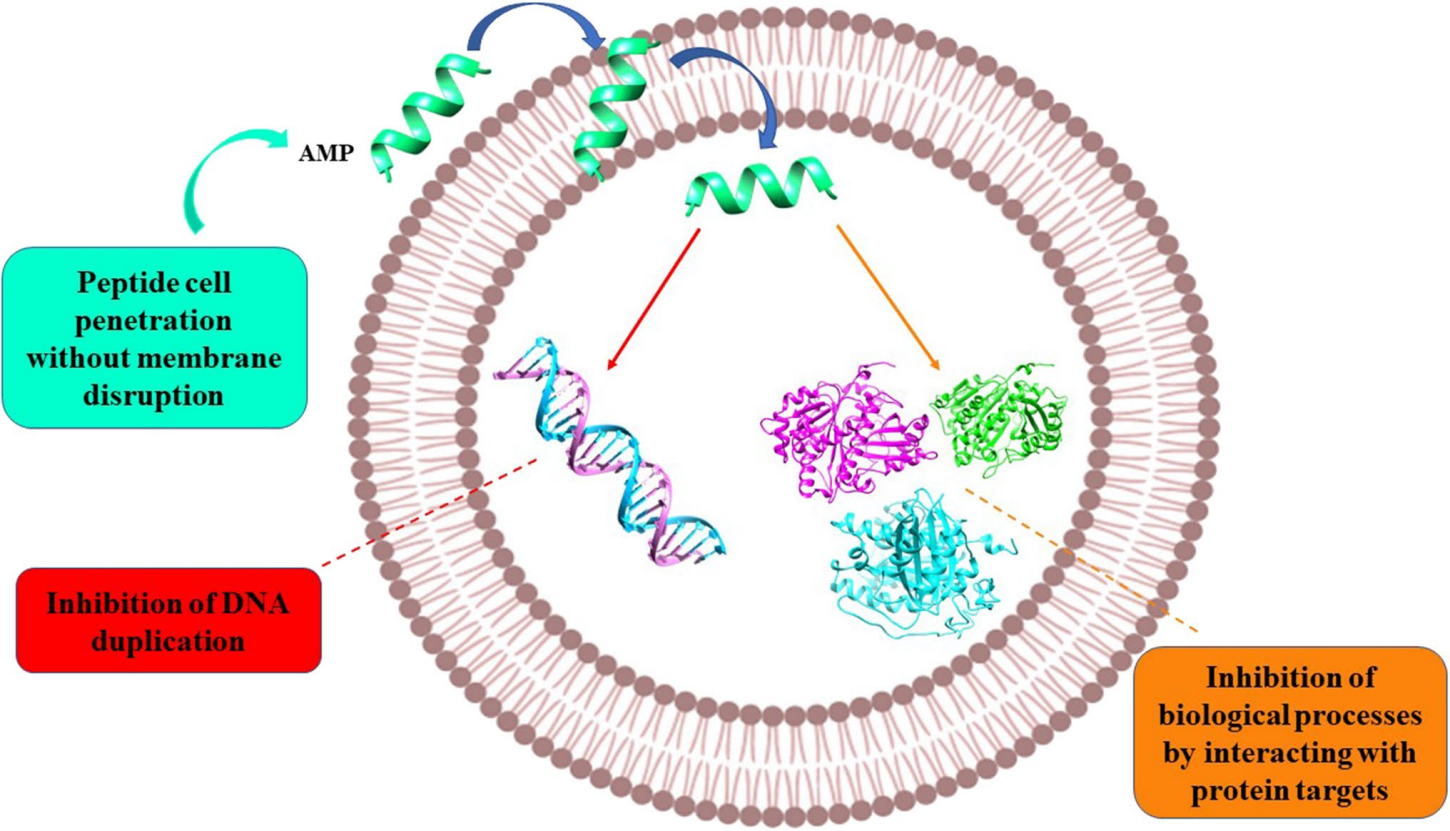
Schematic representation of AMP non-membranolytic mechanism. In this case, AMPs can penetrate into the bacterial cell without membrane break, causing bacterial death by interacting with intracellular targets, including DNA and proteins involved in cellular division or protein synthesis

Most insect AMPs show a positive net charge which allows the interaction with the negatively charged molecules exposed on the bacterial cell surfaces, i.e. LPS of Gram-negative and teichoic acids of Gram-positive bacteria, respectively. Then, the electrostatic attraction is the first interaction that occurs between peptides and cell membranes [86, 171]. Hence, AMPs can generate an unbalancing of ion flows across the membrane (i.e. depolarization). This process consequently produces permeabilization of the bacterial membrane [172]. After reaching the onset concentrations, the formation of pores and the subsequent cell death can be induced (Fig. 10). As demonstrated for other peptides deriving from different organisms, insect AMPs can also act through a non-membranolytic mechanism (Fig. 11) [78]. In this case, AMPs lead to bacterial death by interacting with intracellular targets, as observed, for example, for the Temporin L peptide derived from *Rana temporaria*. It inhibits cell division by binding the FtsZ protein that is the key factor of the divisome complex and is essential in Z-ring formation in *E. coli* [173]. Insect proline-rich peptides are also able to bind other intracellular targets such as the chaperone DnaK or the protein synthesis apparatus [174] (Fig. 11).

We focused our main attention on the mechanism of action of defensins, cecropins and attacins AMPs. Insect defensins may lead to bacterial death through the membranolytic mechanism leading to pore formation on the bacterial membranes or can interact with phospholipids to induce microheterogeneity in the lipid membrane [175, 176] (Fig. 10a). LPS could represent a barrier for the antibacterial activity of insect defensins. Indeed, it has been demonstrated that *E. coli* strains with mutants of LPS are more sensitive to insect defensins [177].

Several studies have been performed to understand cecropin mechanism of action and to identify the functions of specific residues. Most mature cecropins have a tryptophan (Trp) residue in the first or second positions, which confers antimicrobial activity to the peptide [1, 72, 171, 176]. It has been demonstrated that the Trp2 and Phe5 residues in papiliocin peptide, identified in *Papilio xuthus*, are essential for the peptide interaction with LPS in the outer membrane and then for the permeabilization of the inner membrane of Gram-negative bacteria [175].

Although cecropins do not interact with specific receptors, several mechanisms have been proposed to explain the pore formation (Fig. 10). Among these, the carpet model, characterized by high peptide concentration, that leads to the membrane disruption by micelles formation (membranolytic mechanism) (Fig. 10d), is the most accredited. In particular, the interaction via the carpet mechanism assumes that peptides cover the membrane and interact only with the lipid head groups. They associate with the bacterial membrane and then the peptide non-polar side chains fit in the membrane hydrophobic core while the polar residues interact with the lipid phosphates, forming micelles with the fragmented membrane [178, 179]. At low peptide concentrations, cecropins can form channels or pores in specific sections of the membrane [115, 178, 180].

The toroidal pore mechanism, considered as a part of the membranolytic mechanism, consists of peptides insertion, perpendicularly into the bacterial membrane bilayer, a subsequent interaction with the head groups of the lipids to finally induce the bilayer curvature (Fig. 10c). Instead, the barrel-stave pore formation model suggests that the peptides permeate through the bilayer [181] (Fig. 10b). It has been observed that cecropins identified in *H. cecropia* form a barrel (barrel-stave model), which penetrate the bacterial membrane. Concerning peptides shorter than 22 residues they, however, act through a toroidal pore model, in which the pore is composed by both lipids and peptides [178].

Furthermore, several studies described the ability of AMPs to overpass the membrane using a specific interaction with bacterial phosphatidylethanolamine present at higher concentration onto the bacterial membranes [172]. Nonetheless, it is widely accepted that AMPs can target several functions of the bacterial cytoplasm, including the synthesis of nucleic acids, proteins, enzymes, and cell walls. The ability to interfere with several bacterial biosynthetic pathways explain the difficulty in developing resistance towards AMPs [182–184].

Regarding attacin peptides, they can inhibit *E. coli* cells growth by preventing the synthesis of several bacterial porins, which are outer membrane proteins, such as OmpA, OmpC, and OmpF by binding to LPS without penetrating the inner membrane or cytoplasm [139]. Moreover, a well-known peptide, called melittin, is a 26 residues peptide toxin identified in *A. mellifera* venom and is effective against bacteria [185, 186]. It has a strong antibacterial activity against several bacteria and it binds to membrane surfaces leading to pore formation and then to cell lysis [187].

## Stability improvements of peptides against proteolytic susceptibility

### Overview

The main disadvantage of peptide structure is the susceptibility toward both host and microbial proteolytic degradation, that may occur before the AMPs can exhibit the pharmacological effect [188]. Therefore, different strategies have been exploited so far.

Peptide drug candidates must deal with bioavailability and biodistribution issues. In reaching the biophase, AMPs have shown low stability in plasma, low oral bioavailability due to protease susceptibility, and rapid hepatic as well as renal clearances. Biopharmaceutical issues, such as high hydrophilicity and a poor ability to cross physiological barriers, must also be considered. Medicinal chemistry can also help with modifications of the wild-type sequence to improve the poor molecular stability or to modulate the conformational flexibility [189, 190]. For instance, the first studies about drosocin, an O-glycosylated AMP from the fruit fly (i.e. *D. melanogaster*), demonstrated inactivity when injected into *E. coli*-infected mice. Drosocin demonstrated a loss of stability and consequently a loss of antibacterial activity [15, 147]. A series of subsequent studies showed that serum stability of the molecule was improved by considering the non-glycosylated drosocin analogs, which reported an extended half-life in mouse serum and improved activity against Gram-negative pathogens *E. coli* and *K. pneumoniae*. Therefore, drosocin analogs with trans-4-hydroxy-L-proline positions were found to be four- to eight-time more stable in mouse serum than the unmodified analogs [191].

Furthermore, not only the linear and free chain terminations but also the presence of multiple cleavage sites can be readily recognized by the host and bacterial proteases, which can promptly degrade AMPs into inactive fragments. However, after chemical modifications integrated to improve molecular strength against hydrolysis a new structure of AMP is achieved, so it is fundamental to analyze the new AMP characteristics, to avoid, among other issues, bioaccumulation and toxicity [192]. Likewise, the eventual risk of immunogenic effects must be considered [189, 190].

Hence, researchers have to consider structural and functional information such as the study of secondary structure, amino acid composition, length, cationicity, hydrophobicity, and amphipathicity to better obtain a suitable drug candidate with improved stability in vivo.

The chemical modifications play thus a crucial role in the improvement of both the pharmacological activity and biocompatibility, as well as contributing to the chemical stability of the AMP molecules [193–196]. Practically, the main chemical modifications can be included directly during the solid-phase peptide synthesis technique, by which the linear peptide precursor can be assembled from the C-terminal residue, which is linked to the solid resin support. To prevent unwanted couplings during synthesis, a well-known approach considers the use of 9-fluorenylmethyloxycarbonyl (Fmoc) protecting groups. After purification, the obtained linear peptide is let to fold and to cyclize in an appropriate alkaline buffer [197]. Hence, an extra rigidity of the peptide structure may contribute to protract the elimination half-life [198–200].

Including also a broader consideration of the sources, a summary of the most frequent AMP modifications is reported below.

### d-Amino acid substitutions

AMPs can be modified mainly by introducing d-amino acids (DAA) not only in specific regions but also involving all the wild-type sequences. DAA contributes to the partial or the total reversion of the stereochemistry, contributing to enhancing the stability of the peptide against stereospecific proteases [195].

Several studies explored the effect of amino acid substitutions with specific d-amino acids on AMPs activity. To improve the proteolytic resistance, two peptides isolated from the venom of the social wasp *Polybia paulista*, i.e. polybia-MPI and polybia-CP [201], were both partially and totally substituted with DAA. As reported by Zhao and co-workers, the selected AMP was specifically modified with two strategies: (i) by realizing an MPI-analog with d-lysine (d-Lys-MPI); by obtaining (ii) the d-enantiomer of polybia-MPI (d-MPI). Subsequently, the properties of both d-MPI and d-Lys-MPI were compared [202]. Hence, the authors found that the d-Lys-MPI gained extra stability, together with a loss of the antimicrobial activity due to the impairment of the α-helix. On the contrary, to retain the antimicrobial effect as well as to improve molecular stability, the d-MPI demonstrated stable when tested with trypsin and chymotrypsin, and its antimicrobial activity revealed like the wild-type compound (i.e. l-MPI). Against all tested bacteria, the minimal inhibitory concentrations (MIC) of the D-MPI demonstrated greater than the d-Lys-MPI, with an auspicious antimicrobial effect towards both *P. aeruginosa* (MIC 64 μM), and *S. aureus* (MIC 16 μM). Instead, the minimal bactericidal concentration (MBC) was found twofold and fourfold higher, respectively, than MIC values. Taken together, these results have contributed to confirming that the effect of polybia-MPI did not require stereospecific interactions. Therefore, the d-substitution may offer a chemical strategy to improve the stability of the selected APM against protease degradation [202].

The partial and total substitution of DAA in the wild-type sequence was also reported by Jia and co-workers using the polybia-CP compound. Their results demonstrated that both the polybia-CP D-analog, as well as the d-Lys analog, indicated comparable antibacterial activity than the polybia-CP wild-type compound. Furthermore, both MIC and MBC values were not disturbed by each substitution strategy, even though a molecular stability improvement against the enzymatic degradation was observed. Moreover, the D-analog of polybia-CP (D-CP) demonstrated stability to both trypsin and chymotrypsin proteolytic effect, whereas D-Lys analog revealed resistance to trypsin only [203].

However, although preventing protease degradation, partial or total substitution with d-amino amino acids has been demonstrated to be very costly [204].

### N-terminal modifications

The half-life in plasma of a peptide seems related to the typology of the N-terminus residue. N-terminal residues such as Alanine (Ala), Gly, Methionine (Met), Serine (Ser), Threonine (Thr), or Valine (Val) confer to peptides longer half-life. On the contrary, peptides with shorter half-life have been characterized by Arginine (Arg), Aspartate (Asp), Leucine (Leu), Lysine (Lys), or Phenylalanine (Phe) linked at the N-terminus. Likewise, if peptide domains report an enrichment in residues such as Glutamine (Gln), Proline (Pro), Ser, and Thr they are more susceptible to enzymatic degradation [188]. Hence, to block the aminopeptidase action as well as to increase the proteolytic degradation stability of peptides intended for therapeutic use, a common strategy to overpass enzymatic degradation is the acetylation of the N-terminus [195].

Bacteria and eukaryotes can exhibit the N-alkylation of amino acids. For instance, peptides with N-methyl amino acid display an improved resistance against proteolytic degradation, but also a better ability to permeate membranes than their original peptides. N-methyl-amino acids also characterize drugs like cyclosporin A [205]. This cyclic peptide has seven N-methyl-amino acids, and it displays potent bioactivity, and good oral bioavailability [206]. Concerning N-alkylation, Liu and co-workers modified the wild-type sequence of the peptide anoplin in two main ways. They chemically modified the anoplin, the smallest linear α-helical AMP isolated from the venom sac of solitary spider wasps *Anoplius samariensis*, with N-methyl amino acids selecting specific positions, as well as introducing fatty acids with various chain lengths. Initially, the authors underwent anoplin with single and multiple N-methyl amino acid substitutions to determine the cleave sites which are recognized by trypsin and chymotrypsin and, therefore, to confer resistance against peptide degradation. Hence, N-methyl amino acids replaced specific residues of Leu, Ile, Lys, and Arg, identified to be sensitive to enzymatic cleavage. The authors found that the steric burden of the N-methyl amino group influenced the molecule conformation and, consequently, the interaction with peptidases. Further evaluations were also conducted to explain whether the antimicrobial activity was affected, as well. For instance, analogs with single and multiple N-methyl amino acid substitutions demonstrated lacking antimicrobial effect against American Type Culture Collection (ATCC) strains of *E. coli*,* S. aureus*,* B. subtilis*,* P. aeruginosa*, and *K. pneumoniae*. The depletion of antimicrobial activity was influenced by the positions or by the number of amino acids replaced with N-methylation. In addition, further explanations were connected to the decrease of the conformational flexibility and to the removal of potential hydrogen bonding that can occur at specific positions. Nonetheless, a slight increase of MIC values when the N-methyl amino acid replacements pertained to the proximity of the C- or N-terminals was also observed [206]. Subsequently, to enhance the antimicrobial activity, the analogs that showed the high proteolytic stability were chosen for the second chemical adjustment. Hence, the N-terminal was modified by introducing fatty acids with chain lengths ranging from C8 to C14. The antimicrobial activity of the N-methylated lipopeptides with C12/C14 exhibited greater antimicrobial effects against both Gram-negative and Gram-positive bacteria, selecting the C12 compound as the most promising analog. However, the cytotoxicity of N-methylated and C12-analog was also observed, due to the non-selectivity membrane affinity of lipid, inducing hemolytic activity [206].

Zhong and colleagues reported a series of new monomer and dimer peptides that they synthesized by conjugating fatty acids at the N-terminus. The selected AMP was a partial DAA substituted analogue of anoplin. Along with the dimerization of the AMP, the authors showed an alternative method to improve both the anoplin antimicrobial activity and the stability. Moreover, the authors found the lowest MIC when the chain length ranged between C8 and C12. Specifically, a fatty acid chain of C10 showed the lowest MIC towards *P. aeruginosa* ATCC 27853. Against both *S. aureus* species, i.e. ATCC 25923 and MRSA 936, they found a MIC of 8 μM. The dimerization of the helix brought greater MICs for almost all tested species of Gram-negative bacteria. Focusing also on *S. aureus* biofilm inhibition percentage, the authors reported a high rate using a concentration of 2xMIC of peptides characterized by chain lengths of C8, C10, and C12. The biofilm inhibition was then found comparable to unmodified anoplin and polymyxin taken as controls. Considering *P. aeruginosa* species, the authors also found a better rate percentage of inhibition using peptides with the fatty acids chain lengths of C10, and C12, both taken at 2xMIC concentration, whereas using dimers, the effect of inhibition was not significant. The authors also suggested that, in combination with conventional antibiotics, the modified AMPs with a fatty acid chain, along with dimer constitution from modified peptides, may open the way to synergism towards the inhibition of the biofilm formation [207].

### Cyclization and dimerization

Molecular stability represents a crucial requirement for AMPs to be used as new active pharmaceutical ingredients. Several disulfide-rich peptide families, such as plant cyclotides [208] or primate-related θ-defensin [209] display Cys-stabilized structures with a well-defined three-dimensional motif. Focusing on AMP modifications, it is possible to realize the cyclization by three main post-translational methods, i.e. by peptide, lactam, or disulfide bonds. Therefore, by chemical or biological approaches, it is possible to restrict the conformational bend of the peptide structure by introducing some conformational constraints. The modification of the wild-type sequence by cyclization confers rigidity to peptide chains, so the new structural achievement exhibits a minor attitude to be hydrolyzed by proteases [195, 196, 210]. Nonetheless, chemical modifications might also affect the pharmacological effect.

The bioactive conformation of drosocin, a 19-residue proline-rich inducible antibacterial peptide from *D. melanogaster* [147, 211], and apidaecin, a 17-residue from *Apis mellifera* [104] were studied by Gobbo and co-workers. They showed that only the large cyclic dimer of apidaecin moderately retained the antimicrobial activity and the obtained bending of the peptide chain was then not a structural element characteristic of the bioactive conformation of drosocin and apidaecin [148].

To be provided with more information about cyclic and circular peptides, it is possible to consult an open-access database http://www.cybase.org.au/ [212]. Furthermore, most of the approved antimicrobial peptides by the FDA are cyclic structures (e.g. vancomycin, oritavancin, dalbavancin, and telavancin). Due to their higher stability in vivo than that of their linear equivalents, molecular stability represents, therefore, a key factor in reaching the approval [213].

### Insect AMPs to counteract the bacterial biofilm issue

The AMPs used against biofilm act in different way such as (i) the inhibition of planktonic bacteria to adhere to the substrate and the increase the expression regarding motility genes check, (ii) the downregulation of the extracellular matrix synthesis, and (iii) direct bacterial killing. However, most AMP databases consider the AMP antibacterial activity against planktonic bacteria. To fill this gap, Di Luca and co-workers created a database (http://baamps.it/) to address the organization of the AMP antibiofilm activity and to support the antibiofilm study. The antibiofilm field can be considered an emerging research area as reported by Home and Di Luca [214, 215].

Several AMPs, particularly insect cecropins, show the ability to counteract biofilm formation. As reported above, biofilms are a group of microbial cells irreversibly associated to a surface and enclosed in a self-produced matrix, which consists of DNA, polysaccharides, and proteins. It constitutes a barrier that protects bacteria from a variety of chemical, physical, and biological stresses [216]. Biofilms can grow on several surfaces including human skin, teeth, as well as bone and urinary tracts implants, valves, and other artificial implants. When bacteria switch to the biofilm mode of growth, the biomaterial-associated infections are difficult to treat with conventional antibiotic therapies [217]. A crucial problem connected to infections causing respiratory illness is also represented by biofilm development within the lung.

Several studies are reported on insect AMP with antibiofilm effect. Hwang and co-workers focused on a defensin-like peptide derived from the dung beetle *Copris tripartitus*. The authors investigated the antimicrobial and antibiofilm activities of a *C. tripartitus*-derived APM, the coprisin, alone, or in combination with conventional antibiotics. The antibacterial susceptibility testing was conducted against Gram-positive and Gram-negative bacterial strains including *E. faecium* ATCC 19434, *S. aureus* ATCC 25923, *S. mutans* KCTC 3065 from the Korean Collection for Type Cultures (KCTC), two *E. coli* strains, i.e. O-157 ATCC 25922, and ATCC 43895 respectively, and *P. aeruginosa* ATCC 27853. Using the Tissue Culture Plate Method, the antibiofilm activity with a pre-formed biofilm method was tested. A high percentage of biofilm inhibition reported as mean ± SD was found when coprisin was tested against Gram-negative species, ranging between 80.4 ± 4.4% and 86.2 ± 3.3%. The combination of coprisin and ampicillin reporting the highest percentage of biofilm inhibition against *E. coli* O-157 and *P. aeruginosa*, i.e. 90.1 ± 2.9% and 91.7 ± 2.5%, respectively [218].

Chemical synthetized Pro9-3 and Pro9-3D defensins, originated from beetle *Protaetia brevitarsis*, inhibited biofilm formation in *E. coli* and *A. baumannii* in a concentration-dependent manner. Pro9-3 peptide was also modified to increase cationicity and resistance to protease activity, adding Arg to the N-terminus: this modification highly increases the ability to inhibit biofilm formation and to disrupt the mature biofilms, also of MDR strains (MDREC 1229 and MDRAB 12010) [219].

Uropathogenic *E. coli* biofilms are a typical complication of urinary tract infections that contribute to chronicize the disease. Insect AMP cecropin A from *G. mellonella* is able to disrupt planktonic and sessile biofilm cells, alone or combined with the antibiotic nalidixic acid. This finding clearly highlights the high potential of synergistic action between AMPs and classical antibiotics to treat in vivo infection [220].

Very interesting studies showed the antibiofilm performances of a complex mixture of defensin, cecropin, diptericin, proline-rich, and domesticin-like peptides, induced by *C. vicina* immune response after *E. coli* M17 strain infection. In nature, *C. vicina* lives in extremely contaminated areas, rich in bacteria, so this insect innately produces a lot of AMPs simultaneously. The AMP complex displays strong biofilm-breaking activity against human antibiotic-resistant pathogens, such as different strains of *E. coli*, *S. aureus*, *P. aeruginosa*,* K. pneumoniae,* and *A. baumannii*. The inhibition degree is strictly related to specific bacterial strains. The possible synergy between AMP mixture and many common antibiotics (meropenem, amikacin, kanamycin, ampicillin, vancomycin, cefotaxime, clindamycin, erythromycin, chloramphenicol, oxacillin, tetracycline, ciprofloxacin, gentamicin, and polymyxin B) was also evaluated, demonstrating different positive level of interaction in all bacteria, except polymyxin B in *E. coli*. Moreover, this AMP cocktail does not have toxicity to human cells [221, 222].

## AMPs in ongoing clinical trials

Currently, there are still no insect-derived AMP products derived from insects on the biopharmaceutical market [223]. Surely, the insect AMPs may be exploited as an alternative to conventional antibiotics, or a support to synergize their activity.

Although many insect AMPs are synthetized and tested against bacterial strains, few clinical studies are achieved, especially concerning anticancer activity. For example, two peptides from *Oryctes rhinoceros*, the rhinoceros beetle, were successfully tested: the defensin ALYLAIRRR-NH_2_ strongly inhibited MRSA in vivo and in vitro; the d-peptide B, an anticancer peptide, disrupted mouse myeloma cells in vitro with no effects on normal leukocytes [224, 225]. Another AMP molecule, Pierisin-1, AMP from *Pieris rapae*, shows anticancer activity, inducing apoptosis and cytotoxicity in some mammalian cancer cell lines (lung, renal, colorectal, bladder, cervical, and liver) by mono-ADP-ribosylation of DNA [226, 227].

Currently, the clinical use of insect AMPs is really limited because of lacking information concerning bioavailability, instability to proteases, toxicity and side effects [228].

## Conclusions

Insects lack adaptive immunity and base their survival on the production of broad-spectrum AMPs which allow them to create powerful defense mechanisms to counteract infections. In fact, due to the variety of substrates they eat and to the environments in which they live, insects have developed a great variety of responses within the innate immunity. For this reason, with over 1 million described species, they constitute an almost inexhaustible source of biologically active compounds. Several bacteria developed multidrug resistance to modern antibiotics, thus there is a great interest in finding and developing new antimicrobial drugs. Most insect AMPs are cationic due to the presence of basic residues in their amino acid sequences. Thus, they are positively charged at physiological pH, and the positive net charge facilitates their binding to negatively charged microbial surfaces through electrostatic interactions. Thanks to their antibacterial activity and to their ability to be active against fungi, viruses and some cancer cell lines, insect AMPs attract great attention in the biomedical field. In addition, it has been demonstrated that some peptides exhibit an antibiofilm activity and this characteristic makes them good candidates for the use on medical devices to drastically reduce the formation of microbial colonies and biofilm development. The balance of several advantageous parameters from innovative drug delivery systems, along with further chemical stability may confer to AMP-based therapies a suitable potency and biocompatibility. Therefore, considering their broad-spectrum antimicrobial activity, AMPs represent interesting candidates for therapeutic use and will certainly be the object of further research in the future. Moreover, the possibility to use the arsenal of insect AMPs will constitute a great advantage as the management of insects in the laboratory and at higher levels has many advantages: low environmental impact, significantly reduced research cost and time, thanks to the simplicity in breeding them and the high rate of reproduction. In addition, insects breeding overcome ethical problems.
